# Errors in estimated gestational ages reduce the likelihood of health facility deliveries: results from an observational cohort study in Zanzibar

**DOI:** 10.1186/s12913-020-4904-5

**Published:** 2020-01-20

**Authors:** Isabel Fulcher, Kaya Hedt, Stella Marealle, Jalia Tibaijuka, Omar Abdalla, Rachel Hofmann, Erica Layer, Marc Mitchell, Bethany Hedt-Gauthier

**Affiliations:** 1 Department of Biostatistics, Harvard T.H. Chan School of Public Health, Boston, USA; 2 Shuford Program in Entrepreneurship, The University of North Carolina at Chapel Hill, Chapel Hill, USA; 3 D-tree International, Dar es Salaam, Tanzania; 4 Bixby Center for Population, Health, & Sustainability, University of California, Berkeley, USA; 5 Department of Global Health and Social Medicine, Harvard Medical School, Boston, USA

**Keywords:** Maternal health, Mobile health, Gestational age measurement, Estimated delivery date, Health facility delivery, Pregnancy, Tanzania

## Abstract

**Background:**

Most maternal health programs in low- and middle- income countries estimate gestational age to provide appropriate antenatal care at the correct times throughout the pregnancy. Although various gestational dating methods have been validated in research studies, the performance of these methods has not been evaluated on a larger scale, such as within health systems. The objective of this research was to investigate the magnitude and impact of errors in estimated delivery dates on health facility delivery among women enrolled in a maternal health program in Zanzibar.

**Methods:**

This study included 4225 women who were enrolled in the Safer Deliveries program and delivered before May 31, 2017. The exposure of interest was error in estimated delivery date categorized as: severe overestimate, when estimated delivery date (EDD) was 36 days or more after the actual delivery date (ADD); moderate overestimate, when EDD was 15 to 35 days after ADD; accurate, when EDD was 6 days before to 14 days after ADD; and underestimate, when EDD was 7 days or more before ADD. We used Chi-squared tests to identify factors associated with errors in estimated delivery dates. We performed logistic regression to assess the impact of errors in estimated delivery dates on health facility delivery adjusting for age, district of residence, HIV status, and occurrence of past home delivery.

**Results:**

In our data, 28% of the estimated delivery dates were a severe overestimate, 23% moderate overestimate, 41% accurate, and 8% underestimate. Compared to women with an accurate delivery date, women with a moderate or severe overestimate were significantly less likely to deliver in a health facility (OR = 0.71, 95% CI: [0.59, 0.86]; OR = 0.74, 95% CI: [0.61, 0.91]). When adjusting for multiple confounders, women with moderate overestimates were significantly less likely to deliver in a health facility (AOR = 0.76, 95% CI: [0.61, 0.93]); the result moved slightly towards null for women with severe overestimates (AOR = 0.84, 95% CI: [0.69, 1.03]).

**Conclusions:**

The overestimation of women’s EDDs reduces the likelihood of health facility delivery. To address this, maternal health programs should improve estimation of EDD or attempt to curb the effect of these errors within their programs.

## Background

Recent development and expansions of maternal health programs, particularly programs targeting the antenatal period, have sought to improve maternal and neonatal health outcomes in low- and middle-income countries. The effective timing of these antenatal interventions requires accurate estimates of the fetus’s gestational age or the date of delivery [1, 2]. A mother correctly knowing the approximate delivery date will allow them to seek out appropriate antenatal care and make necessary preparations for their delivery [2–4]. Further, as programs increasingly rely on community health workers (CHWs) to visit women throughout their pregnancy to promote health-seeking behaviors, identify danger signs, and encourage health facility delivery [5–8], these CHWs must have a reliable estimate of the delivery date to time these home visits and provide care tailored to the stage of pregnancy.

An abundance of recent research has assessed the accuracy of various gestational age measurement tools, most commonly comparing estimated delivery dates (EDD) based on last menstrual period (LMP) to dating by ultrasound [1, 9–13]. These studies show that both methods are subject to both random and systematic error. For dating by LMP, errors may be caused by poor recall, irregularity of cycle, or hormonal contraceptive use [1]. For ultrasounds, a woman’s EDD depends on the physician, hospital, or timing of the measurement during gestation [14]. Between the two methods, ultrasounds are considered the “gold standard” as they tend to outperform dating by LMP [1, 9–11, 13]. However, as ultrasounds are not available in many low-resource settings, additional research has focused on comparing non-ultrasound alternatives to LMP; these studies have almost unanimously determined that dating by LMP is the best option [1, 11, 13]. Table 1 gives the distribution of preterm (< 37 weeks), term (37–40 weeks), and late or post-term (> 41 weeks) based on gestational age by LMP reported from six recent studies. Importantly, due to differences in study designs and populations, these distributions are not directly comparable, but provide a range of plausible birth distributions from LMP measurements covering a variety of settings.

**Table 1:** Distribution of preterm, term, and post-term delivery by last menstrual period in recent studies

Article	Description of study population	Preterm *< 37 weeks*	Term *37–40 weeks*	Late or Post-term *>* *41 weeks*
Savitz et al. (2002) [14]	Women who attended prenatal care clinics in central North Carolina (*n* = 3655)	12%	76%^a^	12%
Neufeld et al. (2006) [11]	Women from four rural villages in Guatemala (*n* = 171)	2%	96%^a^	2%
Jehan et al. (2010) [1]	Low-to-middle income women in Hyderabad, Pakistan (*n* = 1128)	15%	70%	15%
Khambalia et al. (2013) [2]	Women who delivered in the Northern Sydney Central Coast Health Area, Australia (*n* = 10,243)	5%	81%	14%
Ambrose et al. (2015) [12]	US residents from 33 states in 2012 (*n* = 3,944,480)	11%	83%^a^	6%
Deputy et al. (2017) [13]	PRECONCEPT trial participants in the Thai Nguyen province of Vietnam (*n* = 912)	9%	88%^a^	3%

^a^Included births classified as late term (i.e. “term” was defined in paper as 37–41 weeks)

Although studies of these type have been extremely important for helping healthcare providers understand the direction and magnitude of these errors in EDD, further research is needed in two capacities. First, the way LMP is ascertained in many of these studies may not be feasible for most maternal health programs. For example, in the Vietnam PRECONCEPT trial, women’s date of last menstrual period was recorded on a bi-weekly basis by community health workers until the woman conceived [13]. This will likely yield a more accurate estimate of expected date of delivery than can be expected in most maternal health programs. Secondly, despite the abundance of studies investigating factors related to pre- and postnatal care and health facility delivery, the impact of inaccurate gestational age measurements has received little attention [10, 15–20].

The Safer Deliveries program, implemented by the Zanzibar Ministry of Health with technical support from D-tree International, provides a unique opportunity to investigate the differences in estimated and actual date of delivery in maternal health programs. Additionally, as this program collects a variety of demographic and programmatic information about the women enrolled, one can investigate factors related to and resulting from errors in the EDD. In this paper, we utilize data from the Safer Deliveries program to both describe the reasons for and the negative consequences of these types of errors in order to improve future implementation of this and similar maternal health programs.

## Methods

### Program overview and data collection

In 2015, the maternal and neonatal mortality ratios in Zanzibar, Tanzania were 307 maternal deaths per 100,000 live births and 29 neonatal deaths per 1000 live births, respectively [21]. The Safer Deliveries program aimed to reduce the high rates of maternal and neonatal mortality by increasing the number of pregnant women who deliver in a health care facility and attend prenatal and postnatal check-ups. This program began in 2011 and has been implemented in phases, each phase increasing in scope and scale with the third phase starting in January 2016. As of May 2017, the Safer Deliveries program was active in six (out of 11) districts in Zanzibar on the islands of Unguja and Pemba. The program trains CHWs selected by the Ministry of Health to participate in the program based on their literacy, expressed commitment to the improvement of health, and respectability in their communities.

The CHWs work with community leaders and staff at nearby health facilities to identify and register pregnant women. Typically, a pregnancy is confirmed at the health facility during the first antenatal care visit using a pregnancy test. In the absence of reagent for the pregnant test, missing two consecutive periods and clinical evidence of an enlarged uterus is used to determine pregnancy status. During registration, the CHW meets with the woman, her husband and/or other influential members of the family to discuss and enroll in the program. The CHW visits the woman in her home three times during pregnancy; before 7 months; between 7 and 8 months and again between 8 and 9 months gestational age, to screen for danger signs and provide education, counseling and support to help the woman prepare for a facility delivery. The timing of the visits is based on the estimated delivery date.

The program is supported by a digital platform developed by D-tree International, built using Mangologic software. All CHWs have a mobile app running on a low-end Android smartphone which supports case management and decision support to guide the health worker through each visit. The mobile app guides development of a tailored birth plan based on the woman’s obstetric history and risk factors to identify the most appropriate health facility based on her risk profile. The app then uses the woman’s estimated gestational age to provide tailored messages at the appropriate phase of her pregnancy, screen for danger signs and coordinate referrals to a health facility, calculate and track savings needed for transportation and delivery expenses, and links the woman with a community driver for transportation during delivery. All data collected by the CHWs on the mobile app are synchronized in quasi-real time to the Safer Deliveries server, which is available as raw data and visualized on a program dashboard to support monitoring and programmatic decision-making.

### Study population and variables

This study included women enrolled in the Safer Deliveries program by May 31, 2017 (*n* = 9740) who had a live birth by May 31, 2017 (*n* = 4511). We excluded: 253 women from the newly-added Mkoani district of Pemba Island, 2 women with missing LMP date and EDDs, and 31 women with invalid enrollment times. Our final study population included 4225 women.

We used data collected in the mobile app as part of routine care. At enrollment, the CHW collected demographic and health information to support clinical care for the mother. The CHW used the woman’s Reproductive and Child Health (RCH) card, if available, to record information about the EDD and antenatal care visits. The EDD on the RCH card was ascertained at a facility via last menstrual period or ultrasound, if available at the facility. If the woman does not have an RCH card or there is no EDD, the date of LMP and timing of ANC visits were calculated using self-reported date of LMP and this date was used to calculate an EDD. Although we did not collect information on whether EDD was ascertained by date of LMP or ultrasound, only two Primary Health Care Centers and two hospitals in Unguja have an ultrasound machine available; however, these machines may not be commonly used. Further, only 419 (10%) women reported an ANC visit at one of these health centers, and the distribution of preterm, term, and post-term classifications did not significantly differ from women without ultrasound access at their ANC visit(s) (*P* = 0.834). Due to this, we believe that the vast majority of the delivery dates were calculated based on LMP at an antenatal care or community health worker visit.

The CHW also recorded information about obstetric history at the enrollment and categorized the pregnancy as low, medium, or high risk. Based on the risk category, the woman was advised to deliver at a specific facility and given a target amount of money that she should save for transportation to the facility based on pre-negotiated rates with local drivers participating in the program. At enrollment and each subsequent visit, the CHW collected details about how the woman has prepared for delivery, such as amount of money saved, transportation plan, and number of antenatal visits at a health facility. After the woman delivered, the CHW recorded the date and location of delivery.

### Statistical analyses

Two continuous measures were calculated based on the EDD: (1) difference between actual delivery date (ADD) and EDD and (2) estimated gestational age at delivery (weeks). The difference in delivery dates was coded as a categorical variable with four levels: severe overestimate (EDD was 36 days or more after ADD), moderate overestimate (EDD was 15 to 35 days after ADD), accurate (EDD was 6 days before to 14 days after ADD), and underestimate (EDD was 7 days or more before ADD). The accurate category was defined based on multiple studies that reported actual delivery dates to be accurate within 7 and 14-days of the estimated delivery date due to LMP dating error and/or natural variability in length of gestation [1, 2, 13, 14]. We chose to categorize overestimate as moderate and severe to allow the odds of health facility delivery to vary by severity of LMP misestimation. The cutoff of 36 days for severe overestimation was based on the low proportion (< 5%) of women who were beyond this cutoff (e.g. larger misestimation) in studies that reported the full distribution of LMP dating accuracy [2] or gestational age at delivery [2, 13]. Importantly, this variable captures the difference between when the woman expected to deliver and when that delivery actually occurred regardless of whether the difference was due to an error in estimated delivery dates (gestational age) or a true pre- or post-term birth.

In the first analysis, we investigated differences in demographic and programmatic factors across the four difference in delivery dates categories using Chi-squared tests. The demographic factors included: age category (10–20 years, 21–30 years, 31–40 years, 40+ years), parity category (0, 1, 2–4, 5+), district of residence, HIV status, past home delivery, and risk category. The programmatic factors, updated at the latest CHW visit, included: saving at least 100% of recommended savings for delivery, number of ANC visits, total number of CHV visits, location of delivery, having a CHW postpartum visit within 24 h of delivery, and attending a postnatal care visit.

For the second analysis, we examined if differences in delivery dates affected location of delivery, categorized as health facility delivery versus delivery at home, in the community, or on the way to a health facility. Using logistic regression, we calculated unadjusted and adjusted odds ratios for health facility delivery comparing across difference in delivery date categories with accurate as the reference category. For the adjusted analysis, we included the following potential confounders: age, district of residence, HIV status, and past home delivery. Due to some missing values in the confounding variables, only 4198 women were included in the regression analyses (> 99% of sample). All statistical analyses were performed in Stata V15 (StataCorp, College Station, Texas, USA).

## Results

**Fig. 1:**
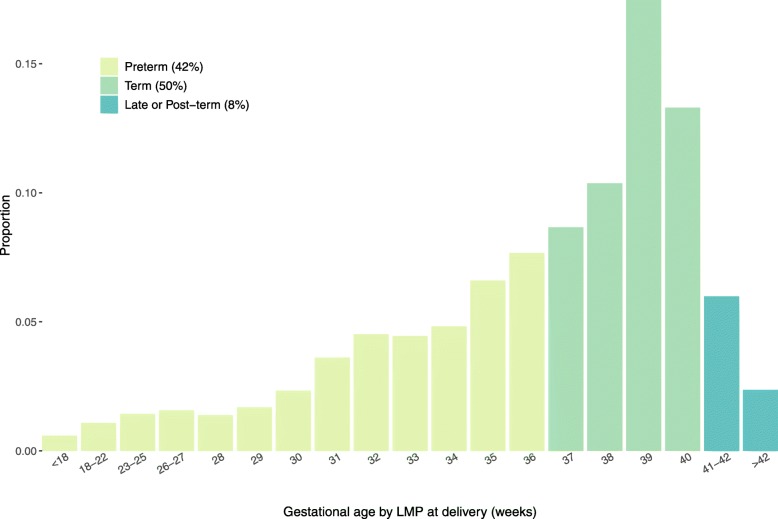
Distribution of gestational age by LMP at delivery (weeks) in the Safer Deliveries program by birth term categorization

Figure 1 gives the distribution of estimated gestation age at delivery (weeks) colored by term categorization [22]. In the Safer Deliveries data, if we classified term of the neonate based on the date of birth relative to the estimated delivery date, then 42% of births would be classified as preterm (< 37 weeks), 50% term (37–40 weeks), and 8% late or post-term (> 41 weeks). For the difference in delivery dates category variable, 28% of the estimated delivery dates were severe overestimates, 23% moderate overestimates, 41% accurate, and 8% underestimates.

In the bivariate analysis, the following demographic factors were significantly associated with difference in delivery dates category: district of residence (*p*-value < 0.001), HIV status (*p*-value < 0.001), and past home delivery (*p*-value = 0.030) (Table 2). Women who live in West and South were less likely to overestimate their delivery date than women living in North A, North B, or Central. Women with unknown HIV status and previous home delivery were more likely to have a severe overestimate in their date of delivery. All of the programmatic factors (Table 3) were significantly associated with the estimated delivery dates category (*p*-values < 0.001). Specifically, women who had severe or moderate overestimates in their delivery dates were less likely to save 100% of recommended savings, attend 4 ANC visits, have 3+ CHW visits, deliver at a health facility, have a postpartum visit within 24 h, and attend a postnatal care visit. Additionally, all of these relationships were starker for women with severe verse moderate overestimates.

**Table 2:** Demographic characteristics by difference in delivery dates category (*N* = 4225)

Variable	Overall	Severe overestimate *36 days or more after ADD*	Moderate overestimate *15 days to 35 days after ADD*	Accurate *6 days before to 14 days after ADD*	Underestimate *7 days or more before ADD*	*p*-value*
Overall		1165 (28%)	969 (23%)	1738 (41%)	353 (8%)	
Age						
10–20	616 (15%)	182 (16%)	147 (15%)	227 (13%)	60 (17%)	0.052
21–30	2409 (57%)	672 (58%)	532 (55%)	1008 (58%)	197 (56%)	
31–40	1092 (26%)	283 (24%)	272 (28%)	446 (26%)	91 (26%)	
41+	108 (3%)	28 (2%)	18 (2%)	57 (3%)	5 (1%)	
Parity						
0	1058 (25%)	277 (24%)	247 (24%)	437 (25%)	97 (27%)	0.133
1	840 (20%)	221 (19%)	210 (22%)	334 (19%)	75 (21%)	
2–4	1658 (39%)	456 (39%)	356 (37%)	711 (41%)	135 (38%)	
5+	667 (16%)	209 (18%)	156 (16%)	256 (15%)	46 (13%)	
*Missing*	*2 (< 1%)*	*2 (< 1%)*	*0 (0%)*	*0 (0%)*	*0 (0%)*	
District						
North A	1012 (24%)	309 (27%)	229 (24%)	384 (22%)	90 (26%)	< 0.001
North B	1425 (34%)	473 (41%)	360 (37%)	511 (29%)	81 (23%)	
Central	699 (17%)	163 (14%)	153 (16%)	298 (17%)	85 (24%)	
West	836 (20%)	167 (14%)	181 (19%)	416 (24%)	72 (20%)	
South	248 (6%)	48 (4%)	46 (5%)	129 (7%)	25 (7%)	
*Missing*	*5 (1%)*	*5 (1%)*	*0 (0%)*	*0 (0%)*	*0 (0%)*	
HIV status						
Negative	4056 (96%)	1095 (94%)	941 (97%)	1676 (96%)	344 (97%)	< 0.001
Positive	89 (2%)	24 (2%)	18 (2%)	36 (2%)	9 (2%)	
Unknown	80 (2%)	46 (4%)	10 (1%)	24 (1%)	0 (0%)	
Past home delivery						
No	2367 (56%)	627 (54%)	549 (57%)	997 (57%)	194 (55%)	0.030
Yes	778 (18%)	255 (22%)	167 (17%)	295 (17%)	61 (17%)	
No past delivery	1058 (25%)	277 (24%)	247 (25%)	437 (25%)	97 (27%)	
*Missing*	*22 (1%)*	*6 (1%)*	*6 (1%)*	*9 (1%)*	*1 (< 1%)*	
Risk category						
Low	2094 (50%)	592 (51%)	469 (48%)	863 (50%)	170 (48%)	0.922
Medium	1355 (32%)	359 (31%)	319 (33%)	562 (32%)	115 (33%)	
High	774 (18%)	212 (18%)	181 (19%)	313 (18%)	68 (19%)	
*Missing*	*2 (< 1%)*	*2 (< 1%)*	*0 (0%)*	*0 (0%)*	*0 (0%)*	

*Calculated using a chi-squared test statistic excluding the missing values included (if applicable)

**Table 3:** Programmatic factors by difference in delivery dates category (*N* = 4225)

Variable	Overall	Severe overestimate *36 days or more after ADD*	Moderate overestimate *15 days to 35 days after ADD*	Accurate *6 days before to 14 days after ADD*	Underestimate *7 days or more before ADD*	*p*-value*
Overall		1165 (28%)	969 (23%)	1738 (41%)	353 (8%)	
Saved at least 100% of recommended savings						
No	1661 (39%)	535 (46%)	390 (40%)	605 (35%)	131 (37%)	< 0.001
Yes	2468 (58%)	603 (52%)	563 (58%)	1087 (63%)	215 (61%)	
*Missing*	*96 (2%)*	*27 (2%)*	*16 (2%)*	*46 (3%)*	*7 (2%)*	
ANC visits						
None	126 (3%)	71 (6%)	15 (2%)	36 (2%)	4 (1%)	< 0.001
1	337 (8%)	195 (17%)	52 (5%)	72 (4%)	18 (5%)	
2	1027 (24%)	391 (34%)	215 (22%)	351 (20%)	70 (20%)	
3	1560 (37%)	345 (30%)	401 (41%)	693 (40%)	121 (34%)	
4	1175 (28%)	163 (14%)	244 (30%)	532 (34%)	325 (40%)	
Total number of CHW visits						
1	1060 (25%)	838 (72%)	161 (17%)	51 (3%)	10 (3%)	< 0.001
2	2208 (52%)	305 (26%)	677 (70%)	1100 (63%)	126 (36%)	
3+	955 (23%)	20 (2%)	131 (14%)	587 (34%)	217 (61%)	
*Missing*	*2 (< 1%)*	*2 (< 1%)*	*0 (0%)*	*0 (0%)*	*0 (0%)*	
Location of Delivery						
At home	690 (16%)	218 (19%)	181 (19%)	241 (14%)	50 (14%)	0.001
On the way	130 (3%)	42 (4%)	27 (3%)	52 (3%)	9 (3%)	
Health facility	3405 (81%)	905 (78%)	761 (79%)	1445 (83%)	294 (83%)	
CHW postpartum visit within 24 h						
No	1781 (42%)	594 (51%)	422 (44%)	623 (36%)	142 (40%)	< 0.001
Yes	2444 (58%)	571 (49%)	547 (56%)	1115 (64%)	211 (60%)	
PNC visit occurred						
No	546 (13%)	218 (19%)	140 (14%)	150 (9%)	38 (11%)	< 0.001
Yes	3679 (87%)	947 (81%)	829 (86%)	1588 (91%)	315 (89%)	

*Calculated using a chi-squared test statistic excluding the missing values included (if applicable)

In the unadjusted logistic model, the odds of health facility delivery were significantly less among women with severe (OR = 0.71, 95% CI: [0.59, 0.86]) and moderate overestimates (OR = 0.74, 95% CI: [0.61, 0.91]) in their delivery dates compared to women with an accurate delivery date (Table 4). For women with an underestimated delivery date, the odds of health facility delivery were about the same as women with an accurate delivery date (OR = 1.01, 95% CI: [0.74, 1.37]). After adjusting for age, district, HIV status, and past home delivery, the odds of health facility delivery among women with moderate overestimates in delivery dates remains the same (AOR = 0.76, 95% CI: [0.61, 0.93]) (Table 5). The result is also similar for women with underestimated delivery dates (AOR = 1.01, 95% CI: [0.73, 1.39]). However, the adjusted odds of health facility delivery among women with severe overestimates moved towards null (AOR = 0.84, 95% CI: [0.69, 1.03]). Note that there is not a significant difference in the odds of health facility delivery comparing women with severe to moderate overestimates in the adjusted model (*P* = 0.361).

**Table 4:** Unadjusted odds ratios for health facility delivery (*N* = 4198)

Variable	Odds Ratio	Standard Error	95% CI	*p*-value
Difference in delivery dates category				
Accurate *EDD is 6 days before to 14 days after ADD*	1 (REF)			
Underestimate *EDD is 7 days or more before ADD*	1.005	0.157	(0.739, 1.366)	0.975
Moderate overestimate *EDD is 15 days to 35 days after ADD*	0.744	0.075	(0.609, 0.907)	0.004
Severe overestimate *EDD is 36 days or more after ADD*	0.710	0.068	(0.588, 0.856)	< 0.001

**Table 5:** Adjusted odds ratios for health facility delivery (N = 4198)

Variable	Odds Ratio	Standard Error	95% CI	*p*-value
Difference in delivery dates category				
Accurate *EDD is 6 days before to 14 days after ADD*	1 (REF)			
Underestimate *EDD is 7 days or more before ADD*	1.005	0.165	(0.730, 1.386)	0.975
Moderate overestimate *EDD is 15 days to 35 days after ADD*	0.757	0.081	(0.614, 0.934)	0.009
Severe overestimate *EDD is 36 days or more after ADD*	0.839	0.086	(0.687, 1.025)	0.086
Age				
10–20	1 (REF)			
21–30	0.778	0.104	(0.599, 1.010)	0.059
31–40	0.711	0.104	(0.534, 0.947)	0.020
41+	0.892	0.265	(0.498, 1.597)	0.700
District				
North A	1 (REF)			
North B	1.035	0.108	(0.843, 1.271)	0.741
Central	0.991	0.124	(0.775, 1.267)	0.942
West	1.673	0.222	(1.290, 2.170)	< 0.001
South	4.107	1.27	(2.237, 7.539)	< 0.001
HIV status				
Negative	1 (REF)			
Positive	0.881	0.256	(0.498, 1.558)	0.663
Unknown	0.267	0.065	(0.165, 0.432)	< 0.001
Past home delivery				
No	1 (REF)			
Yes	0.246	0.022	(0.205, 0.293)	< 0.001

## Discussion

In our study, we would have classified 42% of births as preterm if we believed the estimated delivery dates were correct. Although it has been reported that rates of preterm birth are highest in least developed regions, recent estimates of the preterm birth rates were only 14% in Eastern African countries and 10% in Tanzania [23–25]. Thus, it is likely that the estimated delivery dates recorded by the Safer Deliveries program are incorrect estimated delivery dates. Specifically, the severe and moderate overestimate categories include women who were truly preterm and those with an incorrect estimated delivery date. Due to the large discrepancy in gestational age distributions between the Safer Deliveries program and other studies conducted in this region, we believe the majority of the women in the overestimation categories have incorrect estimated delivery dates.

We found evidence that incorrect estimated delivery dates can negatively impact the likelihood that the mother delivers in a health facility. A 2004 study of the maternal health services available in Kalabo, Zambia, found that women who self-reported that they had adequate knowledge about their estimated date of delivery were 3.7 times more likely to deliver in a health facility than women who did not [3]. Our results were consistent with that finding as women with moderate and severe misestimates were less likely to deliver in a health facility than women with accurate delivery dates.

The distributions of preterm and term deliveries of other studies using LMP to determine estimated delivery date (Table 1) were starkly different than that observed in the Safer Deliveries program, with 42% of Safer Deliveries births categorized as preterm births, compared to 2–15% from the six studies. As the aim of these studies was to assess the performance of various gestational dating methods, greater care was taken to ascertain the date of LMP. This is considerably different than what happens when dating gestational age in practice. For example, in this study, women may not have presented for an antenatal care visit or received a CHW visit until late in their pregnancy creating potential issues with the accuracy of LMP recall. Further, this may have contributed to the systematic overestimation of delivery dates due to a social desirability bias as women may have been less likely to disclose how far along they are in their pregnancy if they were behind schedule for an ANC visit.

We hypothesize the impact of misestimated expected delivery date on delivery in health facilities is related to the woman’s birth preparedness. Previous studies have shown that ill-formed travel plans to the health facility [15, 18, 20, 26], inadequate financial savings for delivery [3, 18, 20, 26], and unexpected delivery [15, 26] were significantly associated with reduced likelihood of health facility delivery. In the context of this study, women with overestimated delivery dates received fewer community health worker visits and attended fewer ANC visits on average than women with an accurate or underestimated delivery date. Due to the fact these visits prepared women for many aspects of delivery, fewer visits may have contributed to less education and counseling for facility delivery preparation. These aspects, and the ability to improve preparedness by improving estimates of EDD, warrant further study.

In order to curb the negative impact of errors in estimated delivery date on health facility delivery, maternal health programs should make program adjustments to either improve EDD estimation and/or alleviate the effects of EDD errors. One option for a program adjustment would be to focus on identifying and enrolling pregnant women in a maternal health program at an earlier gestational age to help with LMP recall [13, 27]. Additionally, community health workers can inform women that the EDD should not be taken as truth and instead expect delivery within a range of plausible dates. To improve estimation of EDD in programs basing estimated delivery dates on LMP, one option is to train CHWs on techniques to aid in women’s recollection of their LMP, as was noted as potentially beneficial in a Guatemala study on dating by LMP [11]. Further, the use of portable ultrasound technology is increasingly viable as this has become relatively inexpensive and could be integrated with the digital technology platform already being used in this setting [28].

Importantly, our findings also indicate that improvement of estimated delivery dates will not increase a woman’s likelihood of health facility delivery if she is truly preterm. However, as a byproduct of more accurate gestational age measurements, classification of preterm and post-term births will improve. This will enable the identification of preterm births at delivery such that neonates can receive appropriate and timely medical care [24, 25]. In addition, the collection of quality data on preterm birth occurrences will enable assessment of the potential risk factors within a population, which can then be used to predict future preterm births in a population.

This study has several limitations that should be considered when interpreting results. Although many confounding factors of the relationship between errors in EDD and health facility delivery were accounted for in the analysis, information was not available on women’s education level and wealth. Several studies based in Tanzania found small or non-significant effects of education level and health facility delivery [16, 17, 26, 29]. However, the reported effects of wealth on facility delivery were inconsistent across studies in East Africa [30]. Further, as this analysis only included women enrolled in the Safer Deliveries program in Zanzibar, Tanzania, the results may not be generalizable to other populations due to both regional differences in women’s accuracy in LMP recall, use of the Islamic vs. Gregorian calendar, and natural variability in gestational duration [2, 11, 13, 31] and structural differences in maternal health programs (i.e. CHW experience, timing of visits, and delivery date estimation). Lastly, we were unable to classify if the births were truly preterm, term, or post-term due to the likely errors in gestational ages and unavailability of birth weight or physical assessments at birth in our data. This would only bias our findings if the decision to go to a health facility differed between women with an overestimated EDD and those who delivered preterm, which we do not believe is the case.

## Conclusions

The distribution of estimated gestational age at delivery in the Safer Deliveries program is substantially different than what is found in previous studies that investigated the accuracy of various gestational age measurement tools. Further, we found evidence that these misestimates can negatively impact the mother’s likelihood of facility delivery. This work provides novel insight into the potential impact of errors in estimated delivery dates and has important implications for the implementation of maternal health programs in low- and middle- income countries. Program implementers should be aware of potential errors in estimated delivery dates and closely monitor the distribution of estimated gestational age measurements in their program’s catchment area. The effects of these errors can be attenuated by program design and/or improvement in the accuracy of gestational age measurements.

## Data Availability

The data that support the findings of this study are available from D-tree International but restrictions apply to the availability of these data, which were used under license for the current study, and so are not publicly available. Data are however available from the authors upon reasonable request and with permission of D-tree International and the Zanzibar Ministry of Health.

## Abbreviations

ADD: Actual date of delivery
ANC: Antenatal care
AOR: Adjusted odds ratio
CHW: Community health worker
EDD: Estimated date of delivery
LMP: Last menstrual period
OR: Odds ratio
